# Rheumatoid Meningitis Presenting With Paraplegia in the Absence of Arthritis: A Case Report and Literature Review

**DOI:** 10.7759/cureus.80359

**Published:** 2025-03-10

**Authors:** Ryoko Muramatsu, Taiki Matsubayashi, Shogo Minomo, Misako Furuki, Masato Obayashi

**Affiliations:** 1 Department of Neurology, National Hospital Organization (NHO) Disaster Medical Center, Tokyo, JPN; 2 Department of Neurology, Nitobe Memorial Nakano General Hospital, Tokyo, JPN

**Keywords:** anti-cyclic citrullinated peptide antibody index, cerebrospinal fluid analysis, magnetic resonance imaging, rheumatoid meningitis, uncomplicated rheumatoid arthritis

## Abstract

Rheumatoid meningitis typically occurs as a late-stage extra-articular central nervous system complication of rheumatoid arthritis (RA); however, its diagnosis can be challenging in the absence of arthritis. A 75-year-old man, without joint pain or stiffness, presented with sudden muscle weakness in both lower limbs. Diffusion-weighted magnetic resonance imaging (MRI) showed hyperintensity along the meninges of the bilateral frontoparietal lobes. He was then diagnosed with rheumatoid meningitis based on the results of the MRI and cerebrospinal fluid (CSF) analysis, which revealed anti-cyclic citrullinated peptide antibody (ACPA) positivity and an increased ACPA index, indicating intrathecal ACPA production. He was then evaluated for uncomplicated RA. Following steroid therapy, his lower limb muscle weakness improved.

This case underscores the importance of considering rheumatoid meningitis as a differential diagnosis in patients presenting with acute-onset paraplegia, even in the absence of arthritis. While the differential diagnosis of acute-onset paraplegia typically includes spinal cord lesions, spinal cord disease was deemed unlikely in this case due to the absence of neck or back pain, sensory disturbances, bladder or rectal dysfunction, and abnormal findings on spinal computed tomography. In this case, the inflammation in the subarachnoid space, as detected by MRI, may have stimulated the bilateral cortex at a high convexity level, resulting in paraplegia. Key diagnostic tools in rheumatoid meningitis include diffusion-weighted MRI, which commonly reveals hyperintensity along the meninges, and CSF analysis demonstrating ACPA positivity and an elevated ACPA index.

## Introduction

Rheumatoid arthritis (RA) is a chronic inflammatory autoimmune disease that primarily affects the joints [1]. Extra-articular manifestations occur in approximately 40% of cases, including rheumatoid nodules, systemic vasculitis, pleuritis, pericarditis, scleritis, and meningitis [1]. Rheumatoid meningitis is a rare extra-articular complication of RA that affects the central nervous system (CNS). Owing to the widespread use of magnetic resonance imaging (MRI), an increasing number of rheumatoid meningitis cases have been identified. While its exact incidence remains unknown, it can be estimated based on the prevalence of RA and the occurrence of rheumatoid meningitis among RA patients. A study based on the Global Burden of Disease study 2017 estimated the global prevalence of RA at 0.27% [2]. Additionally, a recent study in China identified six cases of rheumatoid meningitis among 933 RA patients [3], providing valuable insight into its frequency within RA populations.

Clinically, rheumatoid meningitis presents with a range of neurological and systemic symptoms. A systematic review and meta-analysis reported that focal neurological signs, such as hemiparesis, positive Babinski's sign, aphasia, and hypoesthesia, were the most common symptoms, occurring in 64.28% of cases [4]. Systemic symptoms, including fever, weight loss, malaise, nausea, and vomiting, were reported in 51.78% of cases, followed by episodic headaches (50.00%) and neuropsychiatric symptoms (47.32%).

MRI is a non-invasive biomarker that plays a crucial role in diagnosing rheumatoid meningitis. One of the most sensitive MRI findings in rheumatoid meningitis is hyperintensity along the meninges on diffusion-weighted imaging (DWI) and fluid-attenuated inversion recovery (FLAIR) sequences, often accompanied by contrast enhancement. A previous study of six rheumatoid meningitis cases reported that all exhibited DWI hyperintensity along the meninges [3]. Additionally, in a study of 110 rheumatoid meningitis cases with contrast-enhanced MRI, pachymeningeal enhancement was observed in 60%, while leptomeningeal enhancement was found in 82.72% [4].

Rheumatoid meningitis typically develops in the later stages of RA. However, it can occasionally present before RA is diagnosed. Indeed, 17% of reported rheumatoid meningitis cases occurred before the diagnosis of RA [4]. Diagnosing rheumatoid meningitis in the absence of arthritis is particularly challenging due to its variable and non-specific clinical manifestations [4]. A recent study described the clinical features of rheumatoid meningitis in patients without a prior RA diagnosis, although some of these patients had a history of arthritis [5]. Therefore, the clinical features of rheumatoid meningitis in the absence of arthritis remain unknown. Moreover, the exact pathogenesis of rheumatoid meningitis and the mechanisms underlying its occurrence before the onset of RA remain unclear.

Herein, we report the case of a patient with rheumatoid meningitis presenting with an acute onset of paraplegia but without arthritis. Additionally, we reviewed the clinical features of rheumatoid meningitis in patients without arthritis, including previously reported cases and our own, to enhance our understanding of this disease.

## Case presentation

A 75-year-old man experienced a sudden onset of bilateral lower limb weakness, predominantly on the left side, while attempting to stand. The weakness rapidly worsened within two hours and was accompanied by gait disturbance. His medical history included bronchial asthma, diabetes mellitus, and emphysema. He was taking sitagliptin for diabetes, as well as montelukast, theophylline, tiotropium, and fluticasone/vilanterol for bronchial asthma. He was conscious and alert upon presentation, with the following vital signs: body temperature, 37.5°C; blood pressure, 136/78 mmHg; heart rate, 100 beats/min; and oxygen saturation, 97% in room air. He had no head, neck, or back pain, and did not experience vomiting, photophobia, or any other joint abnormalities, such as swelling, heat, or pain. A cranial nerve examination showed no abnormal findings, but a physical examination showed left-dominant muscle weakness in the lower limbs (manual muscle testing, 4/4). The patellar reflexes in the lower limb were brisk, whereas the other deep tendon reflexes were intact. The pathological reflexes, including Babinski’s and Chaddock’s signs, were negative. Convulsion-like myoclonic movements accompanied by increased muscle tone were observed in the left lower limb. The episode was transient and did not recur. Sensory impairment or bladder and rectal dysfunctions were not noted. The patient also exhibited no signs of meningeal irritation, including nuchal rigidity, Kernig’s sign, and Brudzinski’s sign.

Laboratory tests showed a white blood cell (WBC) count of 8,600/μL with 87.9% neutrophils and a C-reactive protein (CRP) level of 0.85 mg/dL. Treponema pallidum antibody was negative. Immunological tests revealed positive results for the anti-cyclic citrullinated peptide antibody (ACPA) (238 U/mL) and rheumatoid factor (RF) (16 U/mL) and negative results for anti-SS-A/SS-B and anti-neutrophil cytoplasmic antibodies. The levels of matrix metalloproteinase-3 (26.3 ng/mL) and angiotensin-converting enzyme (7.6 U/L) were within their respective normal ranges. A cerebrospinal fluid (CSF) examination showed a slightly increased WBC count (25/3 µL) with 92% lymphocytes, elevated protein level (68 mg/dL), normal glucose level (74 mg/dL), elevated immunoglobulin G (IgG) index (1.15), and ACPA positivity (13.9 U/L). The ACPA index, which is calculated as (CSF ACPA/serum ACPA) ÷ (CSF total IgG/serum total IgG), was elevated to 6.0 (normal <1.3), indicating intrathecal ACPA synthesis. Laboratory parameters in this case were summarized in Table 1.

**Table 1:** Laboratory parameters analyzed in the serum and CSF. ACPA: anti-cyclic citrullinated peptide antibody, CRP: C-reactive protein, CSF: cerebrospinal fluid, IgG: immunoglobulin G, RF: rheumatoid factor, WBC: white blood cell.

	Laboratory parameters	Value (units)	Reference value
Serum	WBC	8,600/μL	4,000-10,000/μL
	Neutrophils	87.90%	40-70%
	Glucose	124 mg/dL	73-109 mg/dL
	CRP	0.85 mg/dL	<0.5 mg/dL
	Treponema pallidum antibody	Negative	Negative
	ACPA	238 U/mL	<20 U/mL
	RF	16 U/mL	<14 U/mL
	Anti-SS-A antibody	Negative	Negative
	Anti-SS-B antibody	Negative	Negative
	Anti-neutrophil cytoplasmic antibodies	Negative	Negative
	Soluble interleukin-2 receptor	541 U/mL	220-530 U/mL
	Matrix metalloproteinase-3	26.3 ng/mL	17.3-59.7 ng/mL
	Angiotensin-converting enzyme	7.6 U/L	8-52 U/L
CSF	Color	Clear	−
	WBC	25/3 μL	0-15/3 μL
	Lymphocytes	92%	40-80%
	Protein	68 mg/dL	15-45 mg/dL
	Glucose	74 mg/dL	40-70 mg/dL
	IgG index	1.15	<0.7
	ACPA	13.9 U/L	−
	ACPA index	6.0	<1.3

**Figure 1:**
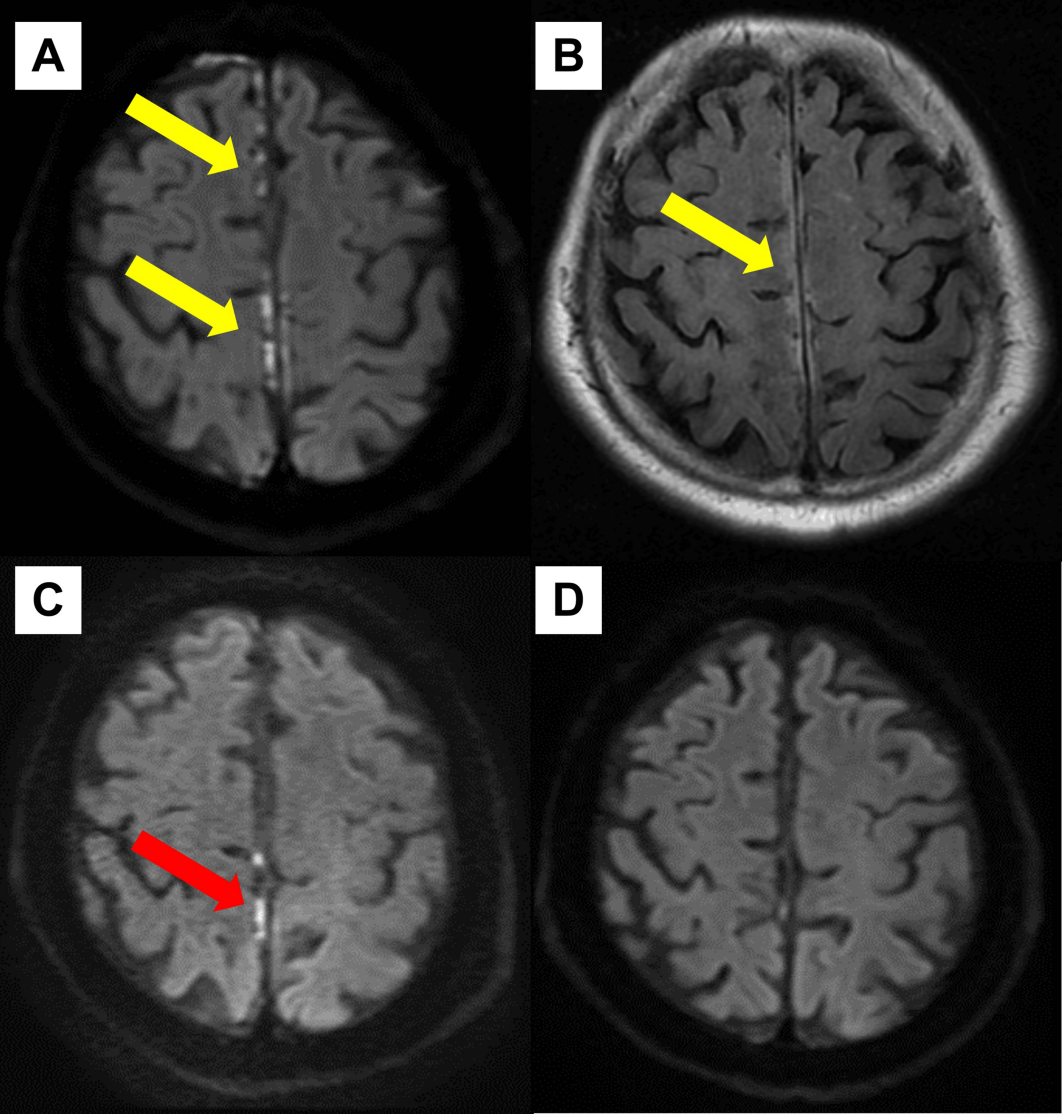
Serial changes in brain MRI findings. On day 1, brain MRI showed hyperintense signals on DWI and fluid-attenuated inversion recovery sequences along the meninges of the right-dominant bilateral frontoparietal lobes (yellow arrows). (A, B) Follow-up DWI-MRI on days 20 and 77 after treatment initiation revealed reduced hyperintensity along the meninges (red arrow) (C) and the disappearance of the findings (D), respectively. DWI: diffusion-weighted imaging, MRI: magnetic response imaging.

Spine computed tomography (CT) showed no abnormal findings, including the presence of tumors, hematomas, or epidural hemorrhage. Brain MRI showed hyperintense signals on DWI and FLAIR sequences along the meninges of the right-dominant bilateral frontoparietal lobes (Figures 1A, 1B). Magnetic resonance angiography revealed no significant stenosis or poor visualization of major intracranial arteries. Contrast-enhanced MRI was not performed because of the patient’s history of bronchial asthma. An electroencephalogram (EEG) was performed, revealing no epileptic discharges; therefore, anticonvulsants were not administered. Additionally, a biopsy was not conducted because of the highly invasive nature of the procedure and his mild symptoms.

He was diagnosed with rheumatoid meningitis based on the exclusion of other diseases, characteristic MRI findings, elevated CSF ACPA level, and increased ACPA index. Blood tests showed positivity for rheumatoid factor and ACPA, but he had no symptoms suggestive of arthritis; he was then evaluated for uncomplicated RA. As treatment for rheumatoid meningitis, high-dose intravenous methylprednisolone (1,000 mg daily for three days) was initiated on day 13 of hospitalization. Six days after treatment initiation, his lower limb muscle weakness improved, and his gait disturbance disappeared. Given the patient's history of diabetes and the potential for worsening glycemic control with prolonged steroid use, we did not initiate oral steroids immediately. He was discharged home on day 20. After careful explanation to the patient, oral prednisolone (10 mg daily) was started on day 33. Prednisolone was tapered without the need for conventional synthetic disease-modifying antirheumatic drugs (csDMARDs).

The hyperintensity along the meninges on MRI was reduced on day 33 (day 20 after treatment initiation) (Figure 1C) and eventually disappeared by day 90 (day 77 after treatment initiation) (Figure 1D). Follow-up CSF analysis on day 90 revealed that the ACPA index had decreased to 3.2. On day 165, prednisolone was discontinued because of his diabetes, and he had no signs of arthritis.

## Discussion

Our case presented with sudden-onset paraplegia with rapid progression in the absence of arthritis as a course of rheumatoid meningitis. Notably, 67% of six rheumatoid meningitis patients exhibited a hyperacute onset within minutes [3], and a case of rheumatoid meningitis mimicking stroke was also reported [6]. Therefore, unlike meningitis of other etiologies, the hyperacute onset, including the sudden onset observed in this case, may be a unique characteristic of rheumatoid meningitis.

To the best of our knowledge, 20 cases of rheumatic meningitis without arthritis at onset have been reported in English and Japanese literature between January 2010 and July 2024 [5,7-23]. The clinical features and laboratory data of rheumatoid meningitis without arthritis at onset are presented in Tables 2, 3, respectively. Previous studies did not specifically report on ethnicity in rheumatoid meningitis. Our literature analysis revealed that the majority of cases were from East Asian countries (Japan, China, Korea), comprising 52.4% (11/21) of the total, followed by cases from Western countries, including the United States and Europe (Germany, Croatia, Ireland, France) (Table 2). However, we acknowledge a potential bias in the data, as our review included both English and Japanese reports. Its most common neurological symptom is hemiplegia, but various symptoms have been observed, including convulsions, headache, psychiatric symptoms, and cognitive impairment (Table 2). Furthermore, some patients, including our case, do not present with fever or headache [8,22,23]. As a result, the absence of specific symptoms can make the diagnosis of rheumatoid meningitis in patients without arthritis symptoms challenging. Notably, our case is the first reported case of rheumatoid meningitis in a patient without arthritis, whose initial symptom was paraplegia.

**Table 2:** Clinical features of rheumatoid meningitis without arthritis at onset. * The year in which each case was reported within the reference. ** The duration from the onset of rheumatoid meningitis to the onset of RA or the final follow-up, shown in parentheses. ***Medications and dosages at the initiation of maintenance therapy. ****Length of time to complete treatment. CPA: cyclophosphamide, DEX: dexamethasone, F: female, M: male, mPSL: methylprednisolone, MTX: methotrexate, NA: not applicable, PSL: prednisolone, RA: rheumatoid arthritis, RTX: rituximab.

Study	Case	Year*	Age (y)	Sex	Ethnicity	Fever	Headache	Neurological symptoms	RA development	Time** (m)	Management		
											Acute	Maintenance***	Duration****
Kim et al. [3]	1	2011	66	M	Korea	NA	NA	Epilepsy, confusion, left paralysis	+	13	−	Oral steroids	6 months
Kawabata et al. [4]	2	2015	69	F	Japan	−	−	Motor apraxia, right paralysis, convulsions	−	(18)	mPSL pulse	Oral steroids	NA
Padjen et al. [5]	3	2015	77	F	Croatia	NA	NA	Right paralysis, convulsions	+	Several	PSL	MTX	NA
Abe et al. [6]	4	2016	84	F	Japan	−	+	Cognitive impairment, apraxia, left paralysis	NA	NA	mPSL pulse	Oral steroids	NA
Magaki et al. [7]	5	2016	37	M	United States	−	+	Right facial weakness, speech difficulty, right hand clumsiness and paresthesia, transient cognitive dysfunction	+	15	PSL	Oral steroids	NA
Shibahara et al. [8]	6	2016	63	M	Japan	+	+	Dizziness, confusion	+	2	mPSL pulse	Oral steroids	NA
Jessee et al. [9]	7	2017	68	F	United States	NA	NA	Confusion, right paralysis, convulsions	+	Several	PSL	MTX	NA
Schuster et al. [10]	8	2018	48	M	Germany	NA	+	Transient left paralysis	−	(20)	mPSL pulse	Oral steroids	2 months
Schuster et al. [10]	9	2018	72	M	Germany	NA	NA	Transient left weakness and numbness	+	15	mPSL pulse	Oral steroids	15 months
Finkelshtein et al. [11]	10	2018	66	M	Israel	NA	+	Transient left lower limb weakness, epileptic seizures	+	13	−		
Lee et al. [12]	11	2019	72	F	United States	NA	NA	Left paralysis, aphasia, mental disorder, epilepsy	NA	NA	mPSL pulse	CPA	4 months
Kira et al. [13]	12	2019	93	M	Japan	−	NA	Unconsciousness, epileptic seizures	−	(8)	Half mPSL pulse	Oral steroids	NA
McKeena et al. [14]	13	2019	59	M	Ireland	+	+	Left paralysis, seizures	NA	NA	mPSL pulse	Oral steroids	NA
Yamaoka et al. [15]	14	2020	62	F	Japan	+	NA	Abnormal behavior, unconsciousness, seizures, cognitive dysfunction	+	3	mPSL pulse	Oral steroids	2 months
Iwao et al. [16]	15	2020	71	M	Japan	+	+	Right lower limb weakness, gait disturbance	−	(1.5)	−	Oral steroids	NA
Rodriguez et al. [2]	16	2020	62	M	United States	+	+	Right lower limb weakness	+	4	DEX pulse	Oral steroids	NA
Chouk et al. [17]	17	2021	62	F	France	NA	+	Sudden unusual sensations, seizures	−	(32)	mPSL pulse	RTX	About 1 year
Matsuda et al. [18]	18	2021	77	M	Japan	−	−	Gait disturbance, communication difficulties	+	17	mPSL pulse	Oral steroids	NA
Yang et al. [19]	19	2024	80	F	China	NA	NA	Transient left paralysis	−	(14)	−		
Yang et al. [19]	20	2024	65	M	China	−	−	Inability to close the left eye, left-side headache	−	(15)	PSL	−	−
Our case	21		75	M	Japan	−	−	Paraplegia, left lower limb convulsions	−	(5.5)	mPSL pulse	Oral steroids	5 months

**Table 3:** Laboratory data of rheumatoid meningitis without arthritis at onset. ACPA: anti-cyclic citrullinated peptide antibody, CRP: C-reactive protein, CSF: cerebrospinal fluid, NA: not applicable, RA: rheumatoid arthritis, RF: rheumatoid factor, WBC: white blood cell, WNL: within normal limits.

Study	Case	Biopsy	Serum			CSF				APCA index
			CRP (mg/dL)	RF (U/mL)	ACPA (U/mL)	WBC (/μL)	Protein (mg/dL)	Glucose (mg/dL)	ACPA (U/mL)	
Kim et al. [7]	1	+	NA	Increased	1448	11	WNL	WNL	NA	NA
Kawabata et al. [8]	2	−	NA	WNL	31.4	47	36.9	NA	Negative	NA
Padjen et al. [9]	3	+	4.33	171.7	405.3	WNL	WNL	WNL	NA	NA
Abe et al. [10]	4	+	WNL	66	1150	7	43	NA	19.3	2.46
Magaki et al. [11]	5	+	4.7	83	>250	16	35	89	NA	NA
Shibahara et al. [12]	6	+	0.4	140	472	37	98	WNL	26.2	NA
Jessee et al. [13]	7	+	NA	208	95.8	8	64	56	NA	NA
Schuster et al. [14]	8	−	WNL	298	>340	300	137	NA	>340	NA
Schuster et al. [14]	9	+	WNL	133	154	51	WNL	NA	NA	NA
Finkelshtein et al. [15]	10	+	WNL	25	266	WNL	WNL	WNL	NA	NA
Lee et al. [16]	11	+	0.9	WNL	197.5	12	25	58	NA	NA
Kira et al. [17]	12	−	2.01	223	306	3	68	64	NA	NA
McKeena et al. [18]	13	+	1.81	88.2	>340	NA	672	46.8	NA	NA
Yamaoka et al. [19]	14	−	WNL	75	30.7	WNL	121	NA	0.7	1.5
Iwao et al. [20]	15	+	0.37	17.9	115	35.7	98.8	NA	17.3	9
Rodriguez et al. [5]	16	+	NA	579	>150	Increased	27.7	70	NA	NA
Chouk et al. [21]	17	−	NA	90	340	20	51	NA	NA	NA
Matsuda et al. [22]	18	+	0.673	29	172.5	60	116	52	172.5	6.4
Yang et al. [23]	19	−	2.85	WNL	>200	15	78	46.8	NA	NA
Yang et al. [23]	20	−	NA	WNL	WNL	WNL	WNL	WNL	NA	NA
Our case	21	−	0.85	16	238	25/3	68	74	13.9	6

A meningeal biopsy is considered the most definitive diagnostic approach for the diagnosis of rheumatoid meningitis [10,16]. Characteristic histopathological findings of rheumatoid meningitis include rheumatoid nodules, vasculitis, and nonspecific meningeal inflammation [24]. However, a study on rheumatoid meningitis reported that 90% of biopsies from ten patients showed only nonspecific inflammation or granulomatous necrosis, making definitive diagnosis challenging [25]. Due to the highly invasive nature of meningeal biopsy, it was not performed in this case. Furthermore, as demonstrated in the present case, rheumatoid meningitis can be diagnosed in the absence of arthritis based on CSF analysis [14,19]. Notably, an elevated ACPA index suggests intrathecal ACPA production [20,22], highlighting the usefulness of CSF biomarkers in the diagnosis of rheumatoid meningitis.

In cases where a biopsy is not performed, as in our case, or when a biopsy fails to yield characteristic findings, excluding other diseases becomes crucial for the diagnosis of rheumatoid meningitis. In this case, it was necessary to differentiate conditions that present with paraplegia as well as those that exhibit high signal intensity along the meninges on brain MRI. To achieve this, we conducted a differential diagnosis based on paraplegia (Table 4) and MRI findings (Table 5). In particular, distinguishing infectious meningitis and tumor-related diseases is essential from both the perspectives of paraplegia and MRI findings. Bacterial or fungal meningitis was considered unlikely, given the patient’s clinical improvement without antibiotic therapy and the presence of normal glucose levels in the CSF. Malignancy was ruled out based on imaging studies, including trunk CT and brain MRI, which showed no evidence of neoplastic processes. In epilepsy, transient post-ictal changes can present as high signal intensity along the cerebral cortex on MRI. However, the resolution of seizures without the use of antiepileptic drugs, combined with the absence of epileptiform discharges on EEG, supports the conclusion that the convulsions were not attributable to epilepsy. Additionally, the differential diagnosis of acute-onset paraplegia commonly includes spinal cord lesions such as spinal cord infarction, myelitis, and atlantoaxial subluxation. In the present case, spinal cord disease was deemed unlikely because there was no evidence of neck or back pains, sensory disturbances, bladder and rectal dysfunctions, or abnormal findings on spine CT.

**Table 4:** Differential diagnoses of this case from the perspective of paraplegia. *Distinct characteristics of the differential diagnoses in this case. **Additional examinations that should be considered as necessary, despite not being performed in this case. CSF: cerebrospinal fluid, CT: computed tomography, ESR: erythrocyte sedimentation rate, MRI: magnetic resonance imaging, MRV: magnetic resonance venography, PCR: polymerase chain reaction, PET: positron emission tomography.

Disease category	Disease	Differential points*	Further examinations**
Infectious diseases	Infectious meningitis	Clinical improvement without antibiotics. Normal CSF glucose.	CSF culture, PCR for common pathogens
	Spinal epidural abscess	No abnormalities on spinal CT.	Spinal MRI
	Spinal tuberculosis (Pott’s disease)	No chronic weight loss or tuberculosis exposure.	Interferon-γ release assay, spinal MRI
Tumor-related diseases	Meningeal carcinomatosis	No abnormalities on spinal CT. Clinical improvement without treatment for the tumor.	CSF cytology, PET-CT
	Meningioma		
	Dural metastasis		
	Spinal cord tumor		
Inflammatory diseases	CNS vasculitis	Negative serum antineutrophil cytoplasmic antibodies.	Brain biopsy
	Neuro-Behcet’s disease	No oral or genital ulcers, or no uveitis.	CSF interleukin-6, human leukocyte antigen-B51
	Sarcoidosis	Negative serum angiotensin-converting enzyme. No findings suggestive of sarcoidosis on trunk CT.	ESR, PET-CT, brain biopsy
	Systemic lupus erythematosus	No systemic symptoms. Negative serum antinuclear antibody, anti-double stranded DNA antibody, or anti-Smith antibody.	ESR
	Rheumatoid meningitis	This case.	ESR, CSF interleukin-6, joint X-ray, brain biopsy
	Idiopathic hypertrophic cranial pachymeningitis	No meningeal thickening on brain MRI.	Contrast-enhanced brain MRI
	Rosai-Dorfman disease	No lymphadenopathy.	Lymph node biopsy
	Transverse myelitis	No sensory deficits or bladder dysfunction.	Spinal MRI
	Multiple sclerosis	No typical lesions on brain MRI.	Oligoclonal band, spinal MRI
Vascular diseases	Dural sinus thrombosis	No signs of venous infarction on brain MRI.	Brain MRV
	Acute aortic dissection	No acute severe chest or back pain.	Contrast-enhanced CT
	Spinal cord infarction	No sensory deficits, bladder or rectal dysfunction, or back pain.	Spinal MRI
	Spinal hemorrhage	No abnormalities on spinal CT.	Contrast-enhanced CT
	Ruptured arteriovenous malformation	No history of sudden severe headaches.	Cerebral angiography
Toxic/Metabolic diseases	Vitamin B12 deficiency	No macrocytosis or neuropathy signs.	Serum vitamin B12
	Neurotoxicity	No exposure history.	Toxicology screening
	Carbon monoxide poisoning		
Other diseases	Posterior reversible encephalopathy syndrome	No hypertensive crisis or eclampsia.	
	Superficial siderosis	No hemosiderin deposition on brain MRI.	
	Atlantoaxial subluxation	No characteristic findings on spinal CT.	Spinal MRI
	Spinal-related diseases		

**Table 5:** Differential diagnoses of this case from the perspective of brain MRI. *Distinct characteristics of the differential diagnoses in this case. Features consistent with rheumatoid meningitis. **Additional examinations that should be considered as necessary, despite not being performed in this case. CSF: cerebrospinal fluid, CT: computed tomography, EEG: electroencephalography, ESR: erythrocyte sedimentation rate, HLA-B51: human leukocyte antigen B51, MRA: magnetic resonance angiography, MRI: magnetic resonance imaging, MRV: magnetic resonance venography, PCR: polymerase chain reaction, PET: positron emission tomography.

Disease category	Disease	Differential points*	Further examinations**
Infectious diseases	Infectious meningitis	Clinical improvement without antibiotics. Normal CSF glucose.	CSF culture, PCR for common pathogens
	Eosinophilic myelomeningoencephalitis	No eosinophilia in CSF or blood.	Spinal MRI
Tumor-related diseases	Meningeal carcinomatosis	No space-occupying lesion and characteristic dural-based lesion on brain MRI. No abnormalities on spinal CT. Clinical improvement without relapse for a long time. No systemic B symptoms. No lymphadenopathy.	CSF cytology, PET-CT, contrast-enhanced brain MRI
	Brain tumor/metastasis		
	Lymphomatoid granulomatosis		
Inflammatory diseases	CNS vasculitis	Negative serum antineutrophil cytoplasmic antibodies.	Brain biopsy
	Neuro-Behcet’s disease	No oral or genital ulcers, or no uveitis.	CSF interleukin-6, HLA-B51
	Sarcoidosis	Negative serum angiotensin-converting enzyme. No findings suggestive of sarcoidosis on trunk CT.	ESR, PET-CT, brain biopsy
	Systemic lupus erythematosus	No systemic symptoms. Negative serum antinuclear antibody, anti-double stranded DNA antibody, or anti-Smith antibody.	ESR
	Rheumatoid meningitis	This case.	ESR, CSF interleukin-6, joint X-ray, brain biopsy
	Idiopathic hypertrophic cranial pachymeningitis	No meningeal thickening on brain MRI.	Contrast-enhanced brain MRI
	Rosai-Dorfman disease	No lymphadenopathy.	Lymph node biopsy
	Temporal arteritis	No temporal headache.	ESR, temporal artery biopsy
Vascular diseases	Dural sinus thrombosis	No signs of venous infarction on brain MRI.	Brain MRV
	Moyamoya disease	No characteristic vascular narrowing on MRA.	Cerebral angiography
Other diseases	Posterior reversible encephalopathy syndrome	No hypertensive crisis or eclampsia.	
	Amyloidosis	No systemic amyloidosis.	
	Transient post-ictal change	No seizure history and normal EEG.	EEG monitoring

Although various mechanisms have been proposed to explain the motor paralysis in rheumatoid meningitis, its exact pathophysiology remains to be elucidated [4]. Interestingly, in the present case, the lower limb with more severe muscle weakness and convulsions was contralateral to the predominant hemisphere showing meningeal hyperintensity. In rheumatoid meningitis, DWI hyperintensity along the meninges is thought to reflect inflammation with a high-density protein component in the subarachnoid space [18,20]. Furthermore, cortical spreading depression caused by inflammation extending from adjacent meninges has been proposed as a potential pathophysiological mechanism underlying focal neurological symptoms in rheumatoid meningitis [3]. Therefore, in our case, inflammation in the subarachnoid space may have induced cortical spreading depression in the right-dominant bilateral cortex at the high convexity level, leading to the development of left-dominant paraplegia and convulsions in the left lower limb.

There is no established consensus on the treatment strategy for rheumatoid meningitis without arthritis. Additionally, whether csDMARDs or continuous maintenance therapy can prevent the development of RA in these patients remains unknown. Among the 21 cases reviewed in our study (Table 2), high-dose steroids were used as acute-phase treatment in 17 cases. For maintenance therapy, steroids were administered in 14 cases, while csDMARDs were used in only three cases. Our patient did not experience a recurrence of rheumatoid meningitis or the onset of RA 5.5 months after the initial symptom. Given that the treatment approach in this case aligns with previously reported cases, it may represent a standard management strategy for rheumatoid meningitis prior to the onset of RA. However, further research is needed to determine the optimal treatment strategy.

ACPA is generated in response to citrullinated peptides, which act as autoantigens [26]. Citrullination is regulated by peptidyl arginine deiminase (PAD), an enzyme with five isoforms [27,28]. Among these, PAD2 and PAD4 have been implicated in the pathogenesis of RA, with PAD2 being highly expressed in the CNS [29]. Our literature review indicates that elevated blood ACPA levels are observed in nearly all reported cases of rheumatoid meningitis, including the present case (Table 3). However, a previous report found that in an RA patient without rheumatoid meningitis, the ACPA index was not elevated despite increased serum ACPA levels [30]. These findings, along with the presence of PAD2 in the CNS and an elevated ACPA index in rheumatoid meningitis, suggest that intrathecal ACPA production may be a specific phenomenon in rheumatoid meningitis. Additionally, ACPA has been shown to induce nociceptive stimulation within the joints by promoting interleukin-8 production and triggering the release of pro-inflammatory cytokines by macrophages [31]. Furthermore, ACPA positivity is associated with an increased prevalence of extra-articular manifestations of RA [31]. Pathological studies of rheumatoid meningitis have demonstrated immune cell and antibody infiltration around small meningeal vessels [4], suggesting that ACPA in the CNS may contribute to immune activation in the meninges. Notably, ACPA elevation is commonly observed prior to RA onset [32]. In conclusion, ACPA produced within the CNS may trigger immune responses in the meninges, potentially leading to the development of rheumatoid meningitis, regardless of RA disease activity.

Serum ACPA has high specificity for RA, regardless of its disease stage [32]. Furthermore, individuals without arthritis but with antibody positivity have a high likelihood of developing RA [10]. At approximately 5.5 months after the onset of rheumatoid meningitis, our patient did not develop RA. However, in previous reports, more than half of the patients without arthritis at the onset of rheumatoid meningitis (10/18) subsequently developed RA (Table 2). Moreover, five out of 10 cases developed RA within one year of the onset of rheumatic meningitis. Therefore, cases of rheumatic meningitis in patients without arthritis, including our case, should be closely monitored for the potential development of arthritis.

In our case, rheumatoid meningitis developed before the onset of RA, suggesting that systemic inflammation might have been minimal. Indeed, among previously reported cases of rheumatoid meningitis without arthritis, CRP levels were within the normal range in five out of 15 cases (Table 3), supporting the possibility that systemic inflammation may not always be prominent in this condition. Furthermore, the role of RF and ACPA in rheumatoid meningitis without a prior diagnosis of RA remains unclear. Interestingly, in our review, three patients were RF-negative but ACPA-positive in serum, whereas no patients were ACPA-negative and RF-positive (Table 3). These findings suggest that ACPA may be a more reliable diagnostic marker than RF in rheumatoid meningitis preceding RA onset. Additionally, CSF ACPA was detected in seven out of eight cases in which it was measured, and the ACPA index was positive in all assessed patients (5/5) (Table 3). These findings indicate that CSF ACPA and the ACPA index may serve as highly sensitive markers for the diagnosis of rheumatoid meningitis. However, their specificity for this condition remains unknown, necessitating further studies to clarify their diagnostic value.

This case highlights several important findings. First, rheumatoid meningitis should be considered a differential diagnosis in patients presenting with paraplegia, even in the absence of arthritis. Second, early MRI and CSF analysis are essential for facilitating timely therapeutic intervention in rheumatoid meningitis. Third, rheumatoid meningitis can occur independently of the duration or severity of RA and may even precede its onset. Finally, close monitoring is necessary for patients with rheumatoid meningitis who do not initially exhibit arthritis, as they may subsequently develop RA.

## Conclusions

We diagnosed a patient with sudden-onset paraplegia with rapid progression, in the absence of arthritis, as having rheumatoid meningitis. The sudden onset with rapid progression observed in this case may be a distinctive feature of rheumatoid meningitis. The diagnosis was primarily supported by DWI demonstrating meningeal hyperintensity on MRI, along with CSF analysis revealing ACPA positivity and an elevated ACPA index. Additionally, the exclusion of other diseases, which is crucial in cases where a biopsy is not performed, further supported the diagnosis. The paraplegia observed in this case may have resulted from cortical spreading depression caused by inflammation in the subarachnoid space of the bilateral cortex at a high convexity level. A literature review further revealed that more than half of the patients without arthritis at the onset of rheumatoid meningitis later developed RA.
